# Multiplexed assays reveal effects of missense variants in MSH2 and cancer predisposition

**DOI:** 10.1371/journal.pgen.1009496

**Published:** 2021-04-22

**Authors:** Sofie V. Nielsen, Rasmus Hartmann-Petersen, Amelie Stein, Kresten Lindorff-Larsen

**Affiliations:** Department of Biology, The Linderstrøm-Lang Centre for Protein Science, University of Copenhagen, Copenhagen, Denmark; St Jude Children’s Research Hospital, UNITED STATES

DNA sequencing plays an increasingly central role in clinical research and diagnostics. Genome-wide association studies have established many links between genes and disease but do not reveal the effect of most of the many possible variants within each disease-related gene. Thus, while the explosion in sequencing of human genomes has revealed millions of missense variants that change protein sequences, we only understand the phenotypic and clinical consequences of a minute fraction of these. This lack of knowledge has direct consequences for clinical action. Even if a variant is discovered in a known disease-related gene, most variants have the status of “unknown significance” (VUS) simply because they have not been encountered before in the population or been studied in the laboratory.

Lynch Syndrome (LS) is a cancer predisposition syndrome that increases the risk of particularly colorectal and gynecological cancers [1]. LS is generally caused by loss-of-function (LoF) variants in one of several mismatch repair (MMR) genes, including *MSH2* [2]. Identification of pathogenic variants in *MSH2* would be of direct clinical relevance, but many missense variants are of unknown pathogenic significance. While computational methods exist to predict pathogenicity, including methods specific for MMR genes [3], they remain imperfect and are only considered as “supporting evidence” for variant classification [4].

For this reason, a number of experimental approaches have been undertaken to assess whether a specific missense variant in *MSH2* is pathogenic or not [5–7]. Some methods can provide detailed mechanistic understanding, yet they can be time consuming since each variant is handled individually and further, they are most easily applied retrospectively. Thus, most current functional assays are challenging to scale to the almost 18,000 possible single amino acid substitutions in MSH2, making it difficult to assign pathogenicity to any new clinically discovered variant.

In contrast, experiments based on multiplexed assays of variant effects (MAVEs; also sometimes known as deep mutational scanning) can be used to probe the effects of thousands of variants in a single experiment [8,9]. MAVEs combine developments in high-throughput DNA synthesis, functional assays, and rapid sequencing techniques. The first step in a MAVE is to construct a DNA library of variants that can be introduced into cells, e.g., by integration on the chromosome, on a plasmid, or by genome editing. The next step is to separate variants by a property of interest. This is often achieved by applying selective pressure, such that cells carrying a functional variant from the library will have higher growth rates than those with nonfunctional variants or, alternatively, by coupling to observable phenotypes like fluorescence followed by cell sorting. The relative frequencies of the variants in the library change depending on how well they are able to perform under selective conditions and are determined by DNA sequencing of the pool of cells before and after the selection. Finally, each variant’s change in frequency is used to compute a score (normalised to wild-type fitness) that quantifies the effect of the variant on the property selected for.

Two recent studies have taken advantage of the MAVE technology to investigate LoF variants in MSH2, assaying two different aspects of MSH2 function [10,11]. Impressively, Jia and colleagues score the function of 94.4% of all possible MSH2 variants, with the goal of identifying missense variants that cause LoF [11]. The assay that they use probes the ability of a given MSH2 variant to mediate G2-M arrest and cell death following treatment with 6-thioguanine (6TG) [12] and has previously been used to classify MSH2 VUS in low throughput [5]. Thus, wild-type-like MSH2 variants will be selected against, and as the study aims to clarify which of the many reported VUS in MSH2 that are potentially pathogenic, it is advantageous that the assay selects for nonfunctional variants. This selection strategy may have proven to be particularly important in this case since 89.4% of all tested variants were characterized as neutral, and MSH2 thus appears highly tolerant to single amino acid substitutions. In fact, 510 out of 934 positions tolerated substitution to any amino acid. In contrast, substitutions to proline or any of the charged amino acids appear to be particularly detrimental, and the majority (77%) of detrimental variants are buried within the native MSH2 structure. Finally, Jia and colleagues compare the ability of the functional scores to classify a curated set of pathogenic and benign variants and find that the experimentally obtained score outperformed several commonly used computational pathogenicity predictors.

The approach taken by Ollodart and colleagues is based on a multiplexed version of a canavanine-resistance assay [13,14], which they use to probe the mutation rate in yeast cells expressing one of ca. 200 different MSH2 variants. They validate their multiplexed experimental setup using a set of variants from a previous study [15], which probed mutation rates for 55 MSH2 variants, one at a time, using a similar canavanine-resistance assay. Finally, they measure mutation rates on a curated set of 185 variants from ClinVar and other clinical sources, which includes benign, pathogenic, and VUS, and find that the assay captures most of these pathogenicity classifications.

**Fig 1:**
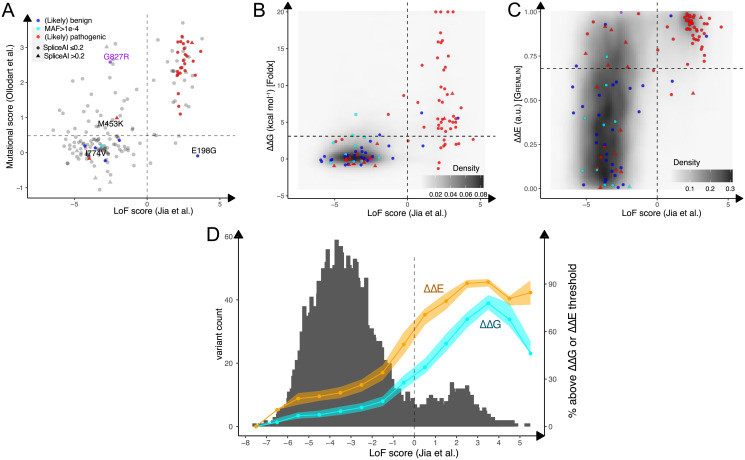
Comparison of MAVE and computational scores for MSH2 variant consequences. (A) LoF scores (on log_2_-scales relative to wild type) from the 6TG assay [11] versus mutation rate fold change [10] for 176 variants probed in both assays with known pathogenic variants shown in red and benign variants in blue; VUS with MAF >10^−4^ [16], and therefore more likely to be benign, shown in cyan. The experimental setups cannot capture effects of potential effects on splicing, so we annotate (triangles) variants where the corresponding change in the DNA is predicted [17] to affect splicing (SpliceAI score >0.2). (B) LoF scores [11] versus ΔΔGs as previously calculated [18] on the MSH2 structure (PDB 2o8e [19]) by FoldX [20]. Variant labels are as in panel A. The gray background density shows the effect of all other variants, with the majority being predicted to have a small effect on stability (ΔΔG<2 kcal/mol) and to be functional in the MAVE. For visual clarity, extremely destabilizing variants (ΔΔGs >20 kcal mol^−1^) are visualized at 20. (C) LoF scores [11] versus ΔΔEs as previously calculated [18] using Gremlin [21] and illustrated in the same way as in panel B. The dashed horizontal line indicates the threshold dividing pathogenic from nonpathogenic variants calculated by ROC analysis as described in [22]. (D) Distribution of MSH2 variants by LoF score (gray) and fraction of variants in each bin that are above the ΔΔG (cyan) and ΔΔE (orange) thresholds of 3.1 kcal mol^−1^ and 0.68, respectively. Shaded areas indicate the error of the fractions arising from uncertainty of the threshold. ΔΔG and ΔΔE values are available at https://github.com/KULL-Centre/PRISM/tree/master/data/proteins/Msh2. 6TG, 6-thioguanine; LoF, loss-of-function; MAF, minor allele frequency; MAVE, multiplexed assays of variant effect; ROC, receiver operating characteristic; VUS, variant of uncertain significance.

The experiments reported by Ollodart and colleagues directly quantify the mutation rates in a mixed cell population but, as of yet, does not scale to the same number of variants as the 6TG survival experiments by Jia and colleagues. The stochastic nature of spontaneously arising mutations makes it challenging to assess in a pooled experiment. The 6TG assay is, on the other hand, specific for MMR proteins, whereas mutation rate measurements are more broadly applicable. Further, the microsatellite instability observed in LS likely reflects an increased mutation rate rather than a failure to signal to G2-M arrest [2]. However, despite differences in both organism and assay, the two analyses largely agree on the functional status of the variants that were probed in both assays (Fig 1A), lending strong support for their use in assigning functional consequences to variants. Of the 176 variants that were assessed by both studies, 86 scored wild-type-like in both, 51 scored as LoF in the 6TG assay and increased mutational rate, and for 39 variants (22%) were there discrepancies between the two studies.

We here briefly discuss the four of these discordant variants with classified phenotypes. E198G is currently classified as benign, and the MAVEs show low mutation rate, but also resistance to 6TG. The variant has previously been shown to have low protein levels and cause functional defects [15,23,24], and E198G is not found in the population sequencing aggregation database gnomAD [16]. G827R showed an increased mutation rate, has been seen as a somatic mutation in a tumor with microsatellite instability, and revision by a clinical expert concluded that the histological phenotype is consistent with pathogenicity. Visual inspection of the MSH2:MSH6 complex (PDB ID 2o8e; [19]) shows that G827 is in the protein–protein interface and that larger and charged side chains such as R would likely perturb binding. The G827R somatic variant was, however, found in a patient that also carries a S676L variant, which may itself be pathogenic [10,25]. M453K is 6TG sensitive but has a moderately increased mutation rate; it has also been described to affect splicing and may be pathogenic for this reason [26]. Finally, I774V scores as wild-type-like in both MAVEs but is listed as pathogenic in ClinVar and is predicted to affect splicing [17]. Variant pathogenicity due to introduction (or removal) of splice sites hence appears to be a notable limitation of the assays used here.

We have previously studied wild-type MSH2 and 24 missense variants including both pathogenic and benign variants as well as variants with unknown pathogenicity [18]. Our results showed that most of the pathogenic variants were found at low steady-state protein levels because they were rapidly degraded by the proteasome. We also found that structure-based computational predictions of the change in thermodynamic stability could be used to predict cellular abundance and thereby pathogenicity for many variants. Together with an assessment of sequence conservation, we suggested that most pathogenic missense variants in *MSH2* cause LS by a mechanism that involves loss of protein stability with resulting loss of abundance and function.

Indeed, 76% of the pathogenic variants that were scored as LoF by Jia and colleagues are predicted to be destabilized beyond the value of ΔΔG = 3.1 kcal mol^−1^ (where ΔΔG refers to the change in protein stability) that we determined by comparing cellular protein levels to stability calculations using the FoldX software [18] (Fig 1B), and 63% of all variants with LoF scores greater than zero (the cutoff determined by Jia and colleagues) have ΔΔG >3.1 kcal mol^−1^. This value can be compared to the just 14% of the functional variants (LoF score <0) that are predicted to have ΔΔG >3.1 kcal mol^−1^. Thus, in line with our previous findings on a small subset of variants, it is likely that a dominant fraction of pathogenic variants has low protein abundance and that this might explain their pathogenicity. In the future, it will be interesting to examine further the 14% of the functional variants that are predicted to be destabilized. Some of these might be explained by inaccuracies in the stability predictions and by the complicated relationship between thermodynamic stability and cellular protein abundance that might mean that the same level of destabilization could result in different cellular abundancies [27]. We also note that there appears to be a continuous transition of LoF and loss of stability, so that while only 7% of the most functional variants (LoF score <−5) have ΔΔG >3.1 kcal mol^−1^, this number increases to 37% among the variants that are regarded as functional but just below the cutoff (LoF score between −1 and 0) (Fig 1D). Indeed, the analysis suggests a gradual LoF beginning from LoF scores > −2, which also happens to be approximately the highest value observed in control synonymous variants [11].

Variants that are stable and abundant in the cell can still cause LoF, e.g., by removing key interactions in the binding sites for DNA, ATP, or other proteins. The effects of stability loss and other mechanisms for LoF can often be quantified by analyzing conservation patterns observed in multiple sequence alignments, for example, through a score termed ΔΔE where large values correspond to nonconservative substitutions. Almost all 6TG-resistant pathogenic variants with high LoF scores have high ΔΔE scores (Fig 1C), indicating that these substitutions are rare or absent among homologs of MSH2 [28,29]. These observations are in line with similar findings in other genes and diseases which show that joint analyses of protein stability effects and sequence conservation may be used both to predict which variants show LoF and to find those that do so due to loss of stability and resulting low protein abundance [30–32]. We also note that 53% of the variants with LoF score <0 and ΔΔG >3.1 kcal mol^−1^ also have high ΔΔE scores, lending further support to their importance to MSH2 function, possibly in aspects that were not captured in the screen, or because these variants show a mild LoF. As for the analysis of ΔΔG described above, we find that variants with high values of ΔΔE become enriched already at intermediate values of LoF score (Fig 1D).

The new results reported by Jia and colleagues and Ollodart and colleagues provide opportunities for future clinical applications. First, the experimental scores—in particular the comprehensive assessment by the 6TG assay—can be useful information for ascribing pathogenicity to new variants that may be discovered in the clinic and might also warrant reassessment of certain previous classifications. Thus, rather than to wait for the results of new functional assays, clinical geneticists may simply look up the variant effects in these experiments if they become validated for clinical use. Second, when more than one variant is present in a patient, it may be difficult to determine from the clinical data which variant(s) is causative, and the data generated by the MAVEs may help in such assignments. Third, data generated by MAVEs are extremely useful for benchmarking prediction methods [29–34], which may in turn be improved for use in other genes and diseases. Fourth, the data and complementary computational analyses may be used to help pinpoint the mechanisms by which variants cause LoF, information that might be particularly relevant for developing future therapies. For example, experiments in yeast have shown that it may be possible to restore function of some MSH2 LoF variants that are unstable and degraded in the cell by disrupting the machinery that recognizes and targets the variants for degradation [35].
